# *Walaphyllium* subgen. nov., the dancing leaf insects from Australia and Papua New Guinea with description of a new species (Phasmatodea, Phylliidae)

**DOI:** 10.3897/zookeys.939.52071

**Published:** 2020-06-09

**Authors:** Royce T. Cumming, Jessa H. Thurman, Sam Youngdale, Stephane Le Tirant

**Affiliations:** 1 Associate researcher, Montréal Insectarium, 4581 rue Sherbrooke, Montreal, Quebec, H1X 2B2, Canada Montréal Insectarium Quebec Canada; 2 PhD Student, Richard Gilder Graduate School, American Museum of Natural History, New York, NY 10024, USA American Museum of Natural History New York United States of America; 3 PhD Student, City University New York, Graduate Center, Ecology and Evolutionary Biology subprogram, New York, NY, USA City University New York United States of America; 4 PhD Student, School of Biological Sciences, University of Queensland, St Lucia, Queensland, Australia University of Queensland Queensland Australia; 5 Los Angeles, California, USA Unaffiliated California United States of America; 6 Collection manager, Montréal Insectarium, 4581 rue Sherbrooke, Montreal, Quebec, H1X 2B2, Canada Collection manager, Montréal Insectarium Quebec Canada

**Keywords:** Biogeography, Djagubay, entomology, Phasmida, Phylliinae, *
Phyllium
*, Queensland, taxonomy, walking leaf

## Abstract

A new subgenus, *Walaphyllium***subgen. nov.**, is described within *Phyllium* Illiger, 1798 to accommodate three leaf insect species. One of the species included is newly described herein as Phyllium (Walaphyllium) lelantos**sp. nov.** from Papua New Guinea. This new subgenus of *Phyllium* can be diagnosed by a following combination of features. This new species is compared to the two additional new subgenus members, *Phyllium zomproi* Größer, 2001 and *Phyllium monteithi* Brock & Hasenpusch, 2003. Also for the first time the male morphology of *Phyllium zomproi* is described and illustrated. To conclude, a brief biogeographical view of the leaf insects on either side of the Torres Strait is presented, as well as a key to species and a distribution map to the known species of Phyllium (Walaphyllium)**subgen. nov.**

## Introduction

**Figure 1.**
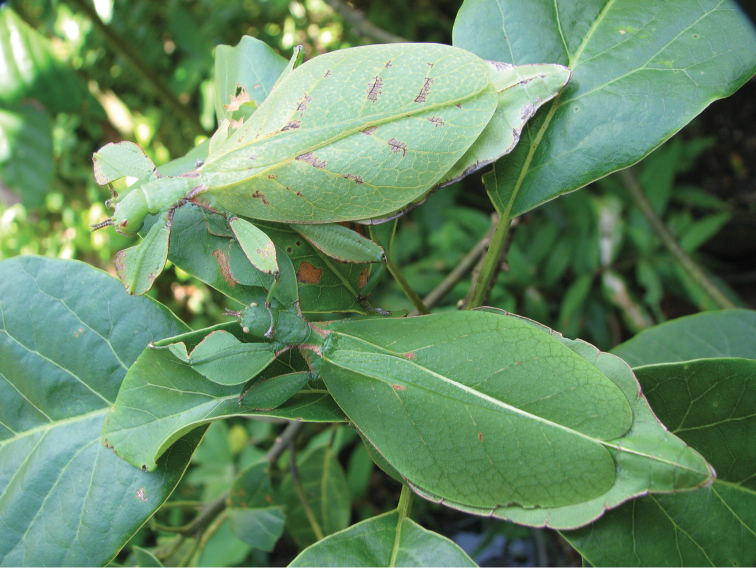
Live *Phyllium monteithi* bred by Jack Hasenpusch in Australia.

**Figure 2.**
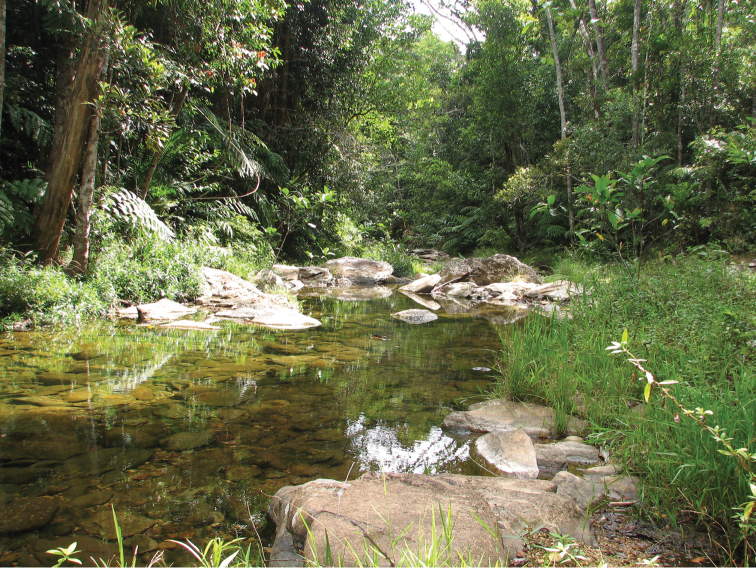
Australia, Queensland, Garradunga, Polly Creek, photo courtesy of Jack Hasenpusch. Lowland mesophyll vine forest, habitat where *Phyllium monteithi* has been recorded.

The leaf insects (Phylliidae) are leaf-mimicking phasmids with flattened abdomens and tibial and/or femoral lobes enlarged to resemble leaves. These adaptations give them incredible camouflage, which allows them to blend in amongst their host plants (Fig. 1). Unfortunately, due to this evolutionary advantageous concealment, specimens are often difficult to locate in their dense jungle habitats and many species of leaf insect are only known from small series or single specimens (Fig. 2). Using the few representatives made available, recent phylogenomic analyses place the Phylliidae as monophyletic within the Old World clade Oriophasmata and sister group to the Bacillidae (Simon et al. 2019).

The Phylliidae in their extant range can be found throughout tropical Asia, from India and China, through to Australia, New Caledonia, and Fiji. Many of these regions where leaf insects are more commonly encountered, such as the Philippines, Indonesia, or India, have had well-known specimen records since the 1700 and 1800’s (Brock et al. 2020). In contrast, records from Australia have only been noted within the the last century with the first written account of Phylliidae in Australia by McKeown (1940). McKeown (1940) speculated that this little-known insect could be native or an accidental introduction from elsewhere in their range (at the time of McKeown he only knew of two leaf insect specimens collected in Australia; McKeown, 1940). It would not be for another 63 years that this species from Australia would gain recognition as an Australian endemic species, *Phyllium monteithi* Brock & Hasenpusch, 2003.

As is often the case, if a phasmid specialist has not spent ample time working within a collection, the Phylliidae specimens within are often misidentified or left with no species-specific identification. This is likely due to the pronounced sexual dimorphism of the leaf insects and the difficulty of collecting specimens because of their natural camouflage. These hindrances often lead to small type series and largely incomplete museum collections, which has historically resulted in the higher taxonomy within the Phylliidae being minimally assessed.

An example of this neglect from in-depth review is the case of the Australian *Phyllium monteithi* which was known for 63 years before it was recognized as a valid and unique species endemic to Australia and not simply *Phyllium siccifolium* as so many specimens have erroneously been called over the years (Brock & Hasenpusch, 2003). Only in recent years have morphological features such as wing venation and egg morphology begun to be extensively reviewed within the Phylliidae (Cumming et al. 2020). Review of these historically ignored features has helped to reveal unique clades, which to date have mostly been concealed when only gross adult morphology is reviewed.

Upon review of the Phylliidae collection within the Natural History Museum United Kingdom, while focusing on wing morphology, an undescribed species of leaf insect from Papua New Guinea was discovered. After reviewing congenerics, we found that not only was this specimen an undescribed species but that there were two other well-known species which formed a unique clade separate from other Phyllium (Phyllium). We here transfer these two species (*Phyllium monteithi* Brock & Hasenpusch, 2003 and *Phyllium zomproi* Größer, 2001), from the *siccifolium* group of Phyllium (Phyllium) as described in Hennemann et al. (2009), and include the herein described *Phyllium lelantos* sp. nov. into their own subgenus Walaphyllium subgen. nov. characterized by the characteristics discussed below.

## Materials and methods

Photographs of the holotype specimen were taken by René Limoges of the Montreal Insectarium using a Nikon D810 DSLR camera with Nikon Micro-Nikkor 200mm f/4 lens on Manfrotto 454 micrometric positioning sliding plate. Lighting was provided by two Nikon SB-25 flash units with a Cameron Digital diffusion photo box. Adobe Photoshop Elements 13 was used as post processing software. Measurements of the holotype were made to the nearest 0.1 mm using digital calipers. The *Phyllium lelantos* sp. nov. holotype specimen is deposited in the Natural History Museum United Kingdom collection. The holotype specimen was loaned to the Montreal Insectarium by the Natural History Museum United Kingdom with the assistance of Judith Marshall and Benjamin Price.

Scanning electron microscope (SEM) images of female *Phyllium monteithi* antennae were produced using a Hitachi Tabletop Microscope (Model: TM4000) at the Centre for Microscopy and Microanalysis (CMM) of the University of Queensland, Australia. All specimens were sputter coated with silver (SPI Module Sputter Coater with Carbon Module, Structure Probe, Inc., West Chester, PA) and mounted on a circular specimen-stage. The specimen in Figure 10B was cleaned using a potassium hydroxide protocol developed by Schneeberg et al. (2017), and allowed to air-dry for 24hours before sputter coating and imaging. This method unfortunately made the antennae brittle and warped after cleaning. The specimens in Figure 10A and C were not cleaned before sputter coating, resulting in subsequent debris.

Egg orientation terminology follows Clark (1978). Wing venation terminology follows Burt (1932) and Ragge (1955).

### Abbreviations

The following institutional abbreviations are used:

**ANIC**Australian National Insect Collection, Canberra, Australia.

**NHMUK**Natural History Museum Natural History, London, United Kingdom.

**QMBA**Queensland Museum, Brisbane, Australia.

**QDPC**Queensland Department of Primary Industries, Indooroopilly, Australia.

**SDEI**Senckenberg Deutsches Entomologisches Institut, Müncheberg, Germany.

**UQIC**University of Queensland, Saint Lucia, Australia.

**Coll JHT** Jessa H. Thurman private collection, Australia.

**Coll RC** Royce T. Cumming private collection, U.S.A.

**Coll SLT** Stéphane Le Tirant private collection, Canada.

The following wing venation abbreviations are used in Figure 5 (listed in order from the anterior to the posterior of the wing):

**C** costa

**Sc** subcosta

**R** radius

**R1** first radius

**R2** second radius

**Rs** radial sector

**R–M** radius to media crossvein

**M** media

**MA** media anterior

**MP** media posterior

**MP1** first media posterior

**MP2** second media posterior

**M–Cu** media to cubitus crossvein

**Cu** cubitus

**CuA** cubitus anterior

**CuP** cubitus posterior

**CuP1** first cubitus posterior

**CuP2** second cubitus posterior

**Cu+1AA** cubitus fused with first anterior anal

**1AA–7AA** anterior anal veins one through seven

**1PA–5PA** posterior anal veins one through five

**1A** first anal

## Taxonomy

### Phyllium (Walaphyllium) subgen. nov.

Taxon classificationAnimaliaPhasmidaPhylliidae

41720270-5C01-5B3E-B0E5-A8FFAB180E06

http://zoobank.org/5416BF7D-0ED2-4825-859D-690662F3FC20

#### Type species here designated.

*Phyllium zomproi* Größer, 2001: 96.

#### Distribution.

**Figure 3.**
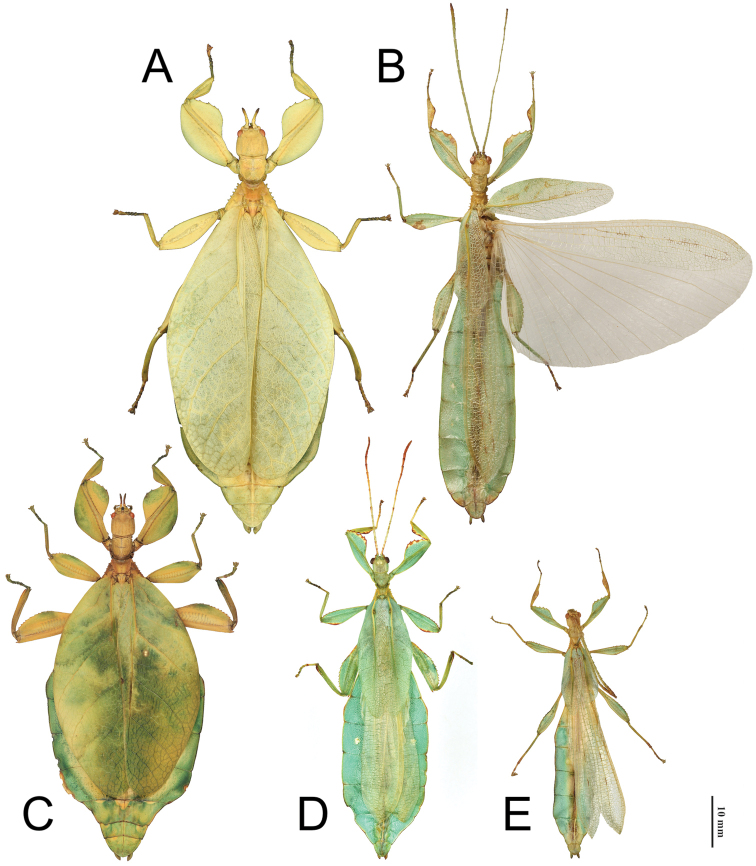
Dorsal view of the known species within the Phyllium (Walaphyllium), scaled to relative size to show the size differences between species **A***Phyllium zomproi* female, Coll SLT**B***Phyllium zomproi* male, Coll RC 17-336 **C***Phyllium monteithi* female dorsal, Coll RC 16-067 **D***Phyllium monteithi* male dorsal, Coll SLT**E***Phyllium lelantos* holotype male, NHMUK. Scale bars: 10 mm.

**Figure 4.**
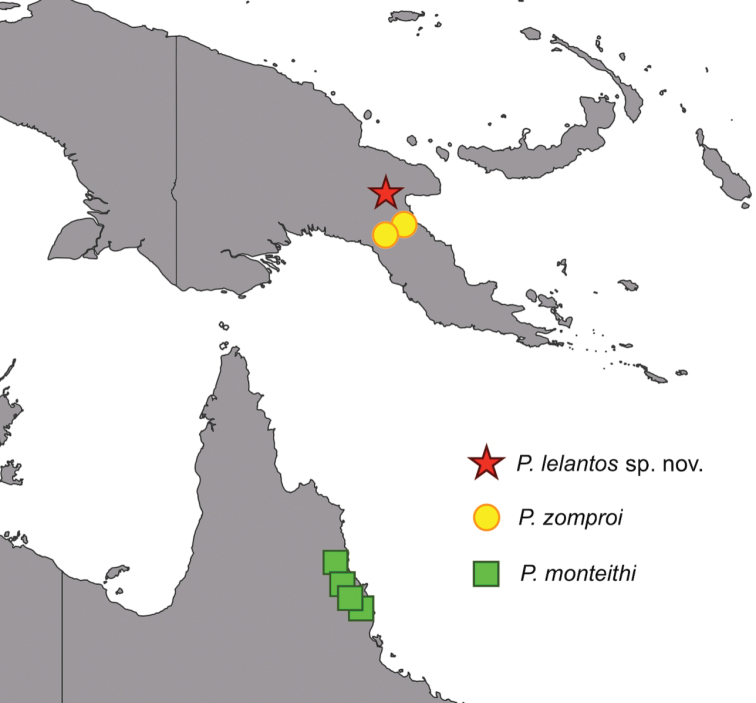
Distribution map for the known species of Phyllium (Walaphyllium) between Papua New Guinea and Australia.

This new subgenus is restricted to Papua New Guinea (two species; Fig. 3A, B, E) and Queensland, Australia (one species; Fig. 3C, D) (Distribution map: Fig. 4).

#### Differentiation.

**Figure 5.**
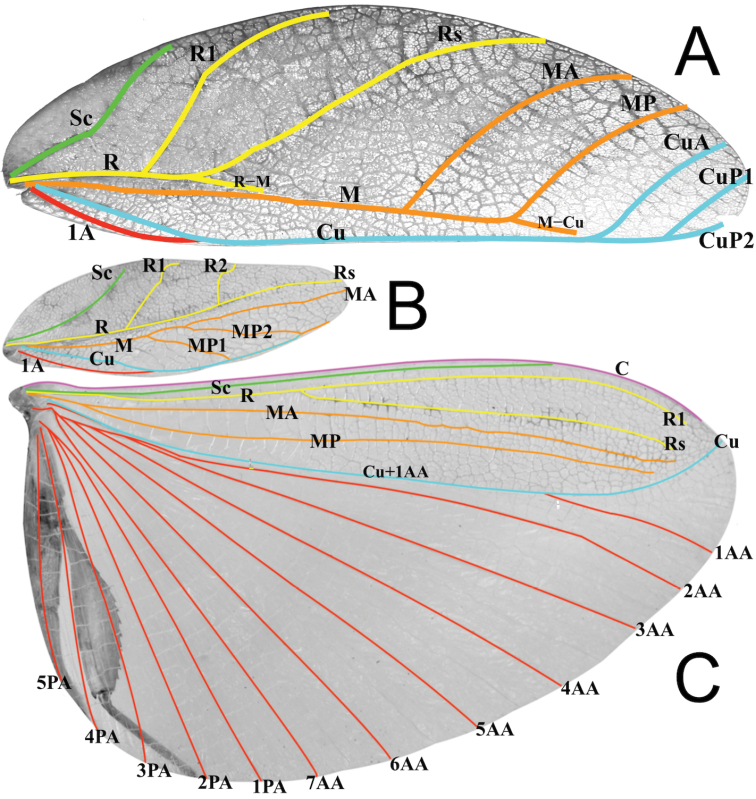
*Phyllium zomproi* wing venation, showing the venation present in the Phyllium (Walaphyllium)**A** female tegmina, Coll RC 18-175 **B** male tegmina, Coll RC 17-336 **C** male alae, Coll RC 17-336.

**Figure 6.**
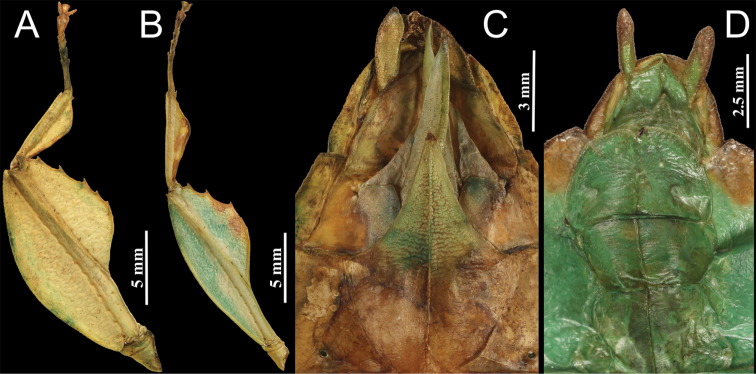
*Phyllium zomproi*. **A** female, profemora and protibia, dorsal, Coll RC 18-277 **B** male, profemora and protibia, dorsal, Coll RC 17-336 **C** female, genitalia, ventral, Coll RC 18-277 **D** male, genitalia, ventral, Coll RC 17-336.

**Figure 7.**
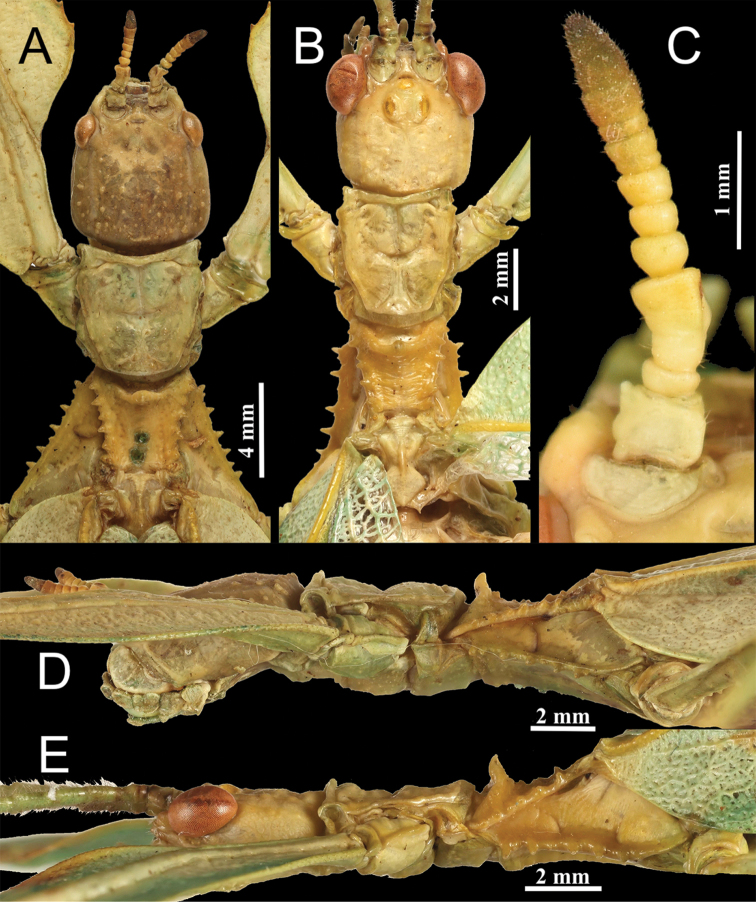
*Phyllium zomproi*. **A** female, head through thorax, dorsal, Coll RC 18-277 **B** male, head through thorax, dorsal, Coll RC 17-336 **C** female, antennae, dorsal, Coll SLT**D** female, lateral, head through thorax, Coll RC 18-277 **E** male, lateral, head through thorax, Coll RC 17-336.

**Figure 8.**
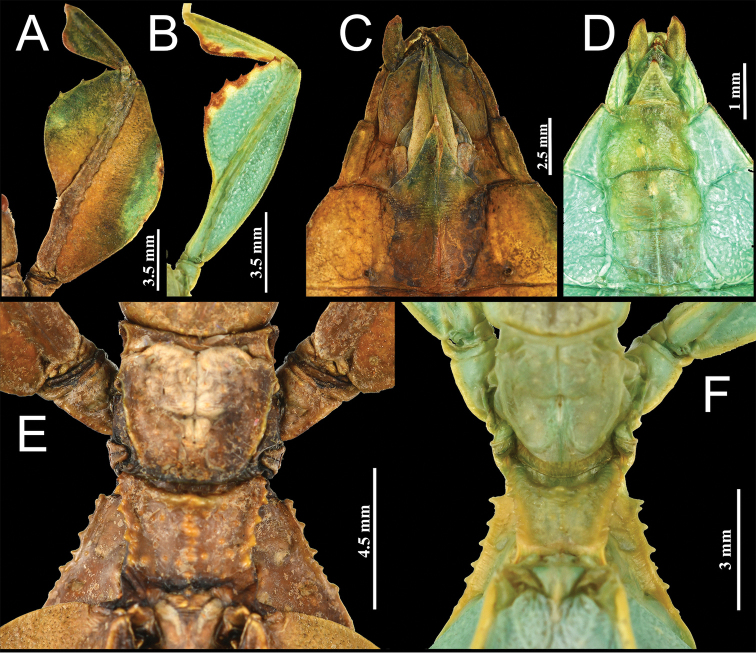
*Phyllium monteithi*. **A** female, profemora and protibia, dorsal **B** male, profemora and protibia, dorsal **C** female, genitalia, ventral **D** male, genitalia, ventral **E** female, thorax, dorsal **F** male, thorax, dorsal. All specimens, Coll SLT.

This new subgenus is easily separated from the other *Phyllium* subgenera by the following combination of features: [Male] tegmina media vein with an anterior media vein (MA) and two posterior media veins (MP1 and MP2) (Fig. 5B) and a vomer with a single apical hook (Figs 6D, 8D, 14C); [Female] tegmina venation with the posterior cubitus split into an anterior cubitus (CuA), first posterior cubitus (CuP1), and second posterior cubitus (CuP2) (Fig. 5A), fourth antennal segment about as long as the following segment individually (Figs 7C, 10A), not short disk-like, and adult females lacking developed alae; [Egg] capsule lacking pinnae, instead with a brittle sponge-textured surface and the operculum conically raised (Fig. 11).

**Figure 9.**
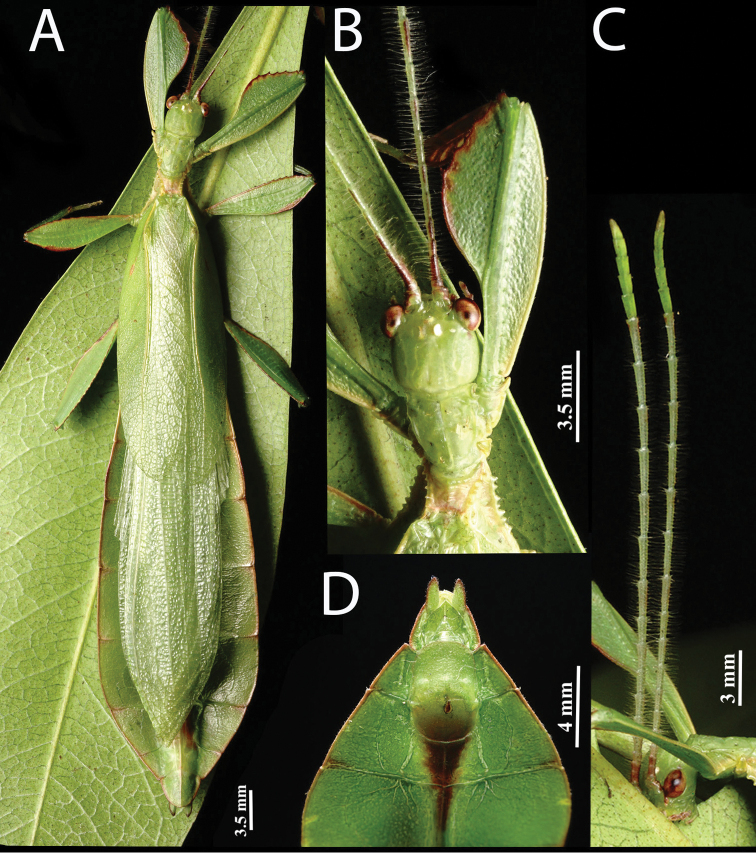
Live male *Phyllium monteithi*, Coll JHT. **A** full body, dorsal **B** detail of profemora, head, and thorax, dorsal view **C** detail of antennae and head, lateral view **D** genitalia detail, ventral view.

Features which characterize this new subgenus, but do not necessarily differentiate it from others are: in males well-developed ocelli (Figs 7B, 9B, 14A); adult abdomen margins parallel (in males) or subparallel (in females) (Fig. 3); tibiae lacking exterior lobes (and only protibial interior lobe developed; Figs 6A, B, 8A, B, 14B); profemoral interior lobe with four to five small serrate teeth in both males and females (Figs 6A, B, 8A, B, 14B); and mesopleurae with five to seven well developed but not large tubercles which are nearly uniform in size throughout the length of the mesopleurae (Figs 7A, B, 8E, F, 9B, 14A). Comparisons to all the *Phyllium* subgenera can be found in Table 1 to summarize distinctive features.

**Table 1.** Morphological comparison of the four recognized *Phyllium* subgenera.

Feature	*Pulchriphyllium* Griffini, 1898	Walaphyllium subgen. nov.	*Phyllium* Illiger, 1798	* Comptaphyllium * Cumming et al., 2019
Egg Capsule	No pinnae (surface pitted and brittle)	No Pinnae (instead an irregular porous, sponge-like texture, brittle, not flexible)	Pinnae (rope, feather, or moss-like of various lengths, all flexible)	Pinnae (feather-like)
Egg Operculum	Conically raised	Conically raised	Flat, margin with rope, or feather-like pinnae or conically raised, with moss-like pinnae	Flat, with a prominent sagittal fan of feather-like pinnae through the center
Male Tegmina (Radial)	Variable: can be branched once into first radius and radial sector, or branched into the first radius, second radius, and radial sector	Variable: can be branched once into first radius and radial sector, or branched as many as four times	Variable: can be branched once into first radius and radial sector, or branched as many as three times	Branched once into first radius and radial sector
Male Tegmina (Media)	With an anterior media vein (MA) and two posterior media veins (MP1 and MP2)	With an anterior media vein (MA) and two posterior media veins (MP1 and MP2)	Variable: can simply be branched into the anterior media (MA) and posterior (MP) or with an anterior media vein (MA) and two posterior media veins (MP1 and MP2)	Branched into the anterior media (MA) and posterior (MP) only
Female Tegmina (Media and Cubitus)	Media and cubitus side by side (touching or or less than one vein width away) until the media posterior diverges to the wing margin	Media and cubitus with spacing between many times wider than the width of a vein for a majority of the length	Media and cubitus with spacing between many times wider than the width of a vein for a majority of the length	Media and cubitus with spacing between many times wider than the width of a vein for a majority of the length
Female Tegmina (Cubitus Branching)	With an anterior and posterior cubitus only	With an anterior cubitus and a branched posterior cubitus into the first and second posterior cubitus	With an anterior and posterior cubitus only	With an anterior and posterior cubitus only
Male Vomer	1 hook	1 hook	1 or 2 hooks	1 hook
Tibial Exterior Lobes (Male and Female)	Yes	No	Yes or No	No

Of these three species in this new subgenus, only *P. monteithi* has been in the phasmid breeding community to date and therefore this is the only species with the newly hatched nymph coloration known. Please see the below *P. monteithi* section for a description of the nymph coloration.

#### Etymology.

*Walaphyllium* meaning “Dancing Leaf”. This subgeneric epithet is a compound of the Latinized name *Phyllium*, the type genus for the family (from Greek φυλλον, -ου (phyllon, -oy) + -um; Poitout 2007), coupled with the prefix *Wala*- which is derived from the indigenous Australian terms “walawalay” used in the Dyirbal language to describe the shake-a-leg dance (Dixon 1972) and “walayi-y,” a verb meaning “to pass by” in the Djagubay language (Patz 1991). Overlapping terms from each of these indigenous languages were investigated as they encompass a large part of the distribution of *Phyllium monteithi*, which is found in the Wet Tropics of far north Queensland, Australia. *Phyllium monteithi* is the most common and well-known species of this new subgenus, thus providing an opportunity to pay respect to the original peoples of this region who may have first appreciated this mysterious insect. This new subgenus is neuter in gender, following *Phyllium*.

#### Species included.

Phyllium (Walaphyllium) zomproi Größer, 2001 (Fig. 3A, B)

Phyllium (Walaphyllium) monteithi Brock & Hasenpusch, 2003 (Fig. 3C, D)

Phyllium (Walaphyllium) lelantos sp. nov. (Fig. 3E)

### Phyllium (Walaphyllium) zomproi

Taxon classificationAnimaliaPhasmidaPhylliidae

Größer, 2001

3C532084-B3C7-508B-BB3A-4874D155F654

Figs 3A, B , 5A–C , 6A–D , 7A–E , 11A–D

#### Distribution.

Papua New Guinea: Morobe Province, Aseki (Winduwe) (NHMUK & Coll RC); Aseki (Wingia) (Holotype: SDEI & Coll RC); Gulf Province (Kaintiba) (Coll RC).

#### Discussion.

Despite being a rarer species in private and museum collections, this is a widespread species, with records from two Papua New Guinea provinces (Morobe and Gulf, Fig. 4). In the original description by Größer (2001) the female and egg were illustrated as well as a subadult male. Since this original description, the adult male has been identified within the NHMUK and within the authors collections. The previously unknown adult male morphology is here described for the first time to help better understand this rarely encountered species.

Body size; females: 80.0–86.0 mm; males: 79.0 mm.

#### Description.

**Male. *Coloration.*** Coloration description based on the limited male specimens from within the authors collections and the NHMUK collection, not on live material. All specimens examined however were with uniform coloration, with little variation between individuals. Overall coloration pale green throughout most of the body. Head through thorax in the preserved specimens is yellow, but this is in all likelihood due to the drying of the specimen and was probably green in life. Compound eyes rusty brown to dark brown color. Antennae pale green or tan, with the terminal segments darker brown. Protibial interior lobe, profemoral interior lobe, and mesofemoral exterior lobe with variable brown patches interrupting the green base color. Tegmina with variable patches of light brown on the green base color, the majority of the tegmina is green. Sclerotized section of the alae can have small patches of brown along the veins, but these are fainter than the brown patches on the tegmina. Abdominal segments V and VI with a small faintly formed clear eyespot of similar width on both the segments. Abdominal segment IX with moderate brown markings on the green, with approximately one third to one half of the segment with brown coloration.

***Morphology.****Head*. Head capsule slightly longer than wide, vertex is lumpy with a smooth texture, not overly granular. Frontal convexity stout with a broad point, apex marked with five to seven thin setae. The posteromedial tubercle is not prominent, only slightly raised from the posterior of the head capsule. *Antennae*. Antennae consist of 22–23 segments (including the scapus and pedicellus), all segments except the scapus and pedicellus and terminal six are covered in moderately dense tan setae that are as long as the antennae segment is wide. The terminal six segments are covered in tan setae that are about as dense as the setae on the other segments but much shorter (some only slightly raised above the segment surface), and the scapus and pedicellus are without setae. Compound eyes large (taking up about half of the head capsule length) but not notably protruding away from the head (Fig. 7B). Three well-developed ocelli are between the compound eyes and slightly raised above the head capsule (Fig. 7B). Antennal fields slightly wider than the scapus width, not notably large (Fig. 7B). *Thorax*. Pronotum with an anterior margin that is clearly but not strongly concave; lateral margins that are straight for the anterior two-thirds and then clearly angled toward the posterior margin which is slightly more than half of the width of the anterior margin and slightly convex (Fig. 7B). Anterior and lateral margins of the pronotum with distinct rims, and the posterior margin lacks a well-developed rim. Face of the pronotum has a lumpy texture and is marked by a distinct sagittal furrow and crescent shaped pit in the center (Fig. 7B). Prosternum is moderately granulose with nodes throughout of relatively even size. Mesosternum surface mostly wrinkled, not as much granulation as the prosternum. Prescutum about as wide as long, with lateral margins slightly converging to the posterior. Lateral rims with six to seven tubercles of slightly varying size and uneven spacing, with most tubercles prominent. Prescutum crest along the sagittal plane with four or five small nodes. The surface of the prescutum is mostly wrinkled in texture and not prominently raised along the sagittal plane. Prescutum anterior margin marked with a prominent slightly recurved tubercle rising above the surface of the pronotum (Fig. 7E). Mesopleurae narrow, nearly parallel for most of their length, only starting to more strongly diverge after about two thirds of the way through with the posterior the broadest portion (Fig. 7B). Lateral margin with five major tubercles throughout the length, and generally two or three small minor tubercles interspersed (Fig. 7B). Face of the mesopleurae smooth except for two distinct pits, one on the anterior third and one on the posterior third. *Wings*. Tegmina moderate in length, extending about one quarter of the way into abdominal segment IV. Tegmina wing venation (Fig. 5B): the subcosta (Sc) is the first vein, running smoothly to the margin and is the first to terminate on the wing margin, about two fifths of the way through the overall tegmina length. The radius (R) spans the entire length of the tegmina with the first radius (R1) branching approximately one third through the length, then a second radius (R2) branches approximately two thirds of the way through the length, and the radial sector (Rs) terminates at the wing apex. The media (M) also spans the entire length of the tegmina (as the media anterior MA, terminating at the wing apex). There are two posterior media veins, the first posterior media (MP1) branches near the middle and meets the cubitus at the wing margin and terminates. The second posterior media (MP2) branches after the first posterior media at about the midline and meets with the cubitus near the wing margin. The cubitus (Cu) runs along most of the tegmina margin and terminates past the midline upon meeting the second media posterior. The first anal (1A) vein terminates upon reaching the cubitus proximal to the midline. Alae well developed in an oval fan configuration, long, almost reaching to the posterior margin of abdominal segment VIII. Alae wing venation (Fig. 5C): the costa (C) is present throughout the entire foremargin giving stability to the wing. The subcosta (Sc) spans approximately three quarters of the wing length running alongside the costa vein the entire length. The radius (R) spans nearly the entire wing length and branches approximately two fifths of the way through into the first radius (R1) and radial sector (Rs). These run gently diverging through about half of their length after which they become parallel and start to bend toward the media vein. Instead of terminating at the wing apex or meeting the media vein, they simply thin out and end individually just shy of the apex. The media (M) branches almost immediately into the media anterior (MA) and the media posterior (MP) which run parallel or subparallel with each other throughout their entire lengths. Neither the media anterior or posterior terminate at the wing apex and like the radial veins the media veins simply thin out and terminate just shy of the apex near where the radial veins terminated. The cubitus (Cu) runs unbranched and terminates at the wing apex bending towards the terminated radial and media veins but not fusing with any. Of the anterior anals, the first anterior anal (1AA) fuses with the cubitus at the wing base and does not diverge from the cubitus until three quarters of the way through the wing length where it diverges away from the curving cubitus until the first anterior anal terminates at the wing margin. The anterior anals two through seven (2AA–7AA) have a common origin and run unbranched in a folding fan pattern of relatively uniform spacing to the wing margin. The posterior anals (1PA–5PA) share a common origin separate from the anterior anals and run unbranched to the wing margin with slightly narrower spacing between them than the anterior anals. *Abdomen*. Abdominal segments II through III only slightly diverging, IV through VII about half as long as wide and with parallel margins giving the abdomen a long boxy appearance. Segments VIII and IX with smoothly rounded margins converging to segment X which is about half as wide as segment IX and with margins that converge more strongly to the apex. *Genitalia*. Poculum starting two thirds of the way through abdominal segment VIII, broad with rounded margins, and ending in an apex that slightly passes the anterior margin of segment X and has a distinct cleft in the center (Fig. 6D). Cerci long and slender with at least half of their length protruding from under the terminal abdominal segment, margins slightly cupped, surface covered throughout in thin setae and a granular surface. Vomer not particularly broad, with nearly straight sides evenly converging to the single apical point that hooks upwards into the paraproct (Fig. 6D). *Legs*. Profemoral exterior lobe smoothly arcing evenly end to end and at its widest is only slightly thinner than the interior lobe. Profemoral exterior lobe margin lacking teeth but does have a slightly granular surface with minimal short setae throughout (Fig. 6B). Profemoral interior lobe rounded without a strong angle and marked with three to four prominent serrate teeth with wide looping gaps between each tooth, the gap in the center is only slightly wider than the gaps on each side (Fig. 6B). Mesofemoral exterior lobe arcs end to end with a distinct bend on the distal third which marks the widest portion of the lobe, no teeth present on the exterior lobe. Mesofemoral interior lobe thinner than the exterior lobe, smoothly arcing from end to end without a distinct bend and with six serrate teeth on the distal half only. Metafemoral exterior lobe without serrate teeth and not broad, straight along the metafemoral shaft. Metafemoral interior lobe wider than the exterior, gently arcing with ten to eleven small serrate teeth throughout the distal three quarters of the length. Protibiae lacking exterior lobe, interior lobe reaching end to end in a rounded scalene triangle, broadest on the distal end (Fig. 6B). Meso- and metatibiae simple, lacking lobes completely.

### Phyllium (Walaphyllium) monteithi

Taxon classificationAnimaliaPhasmidaPhylliidae

Brock & Hasenpusch, 2003

69A726D1-E214-5300-9CA7-FE65849E3095

**Figure 10.**
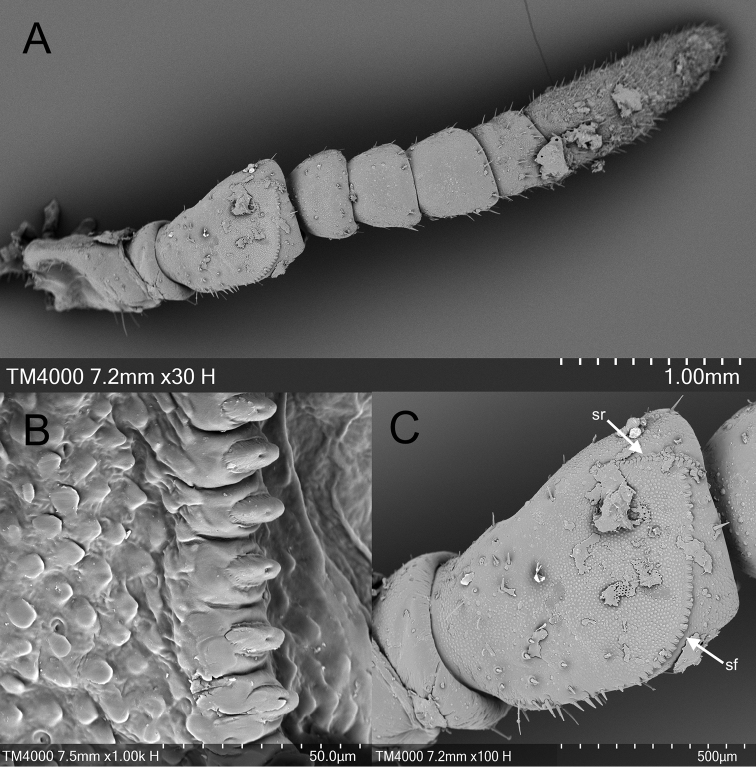
SEM image of female *Phyllium monteithi* antennae, Coll JHT. **A** full antenna **B** details of the stridulatory file of the third antennomere **C** antennomere III in full. Abbreviations: sf = stridulatory file; sr = stridulatory ridge.

**Figure 11.**
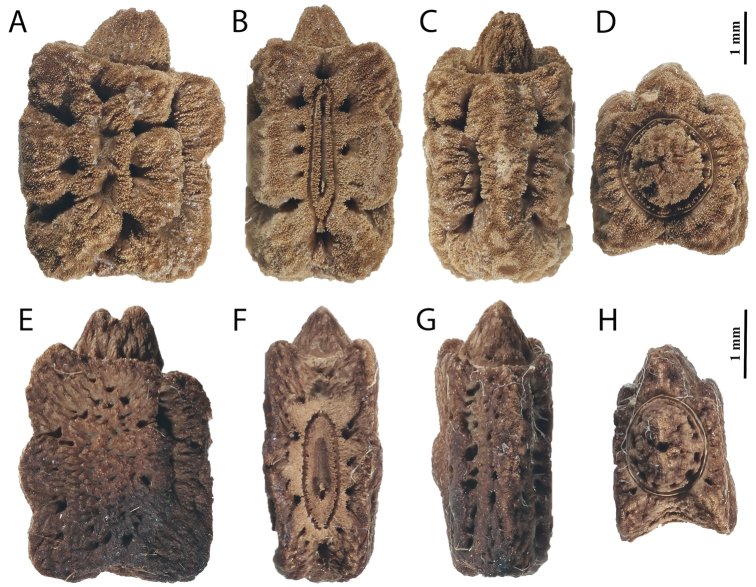
Known eggs for the Phyllium (Walaphyllium). **A–D***P. zomproi*, (Coll RC 19-161) **A** lateral **B** dorsal **C** ventral **D** opercular (anterior) **E–H***P. monteithi*, (Coll RC 17-289) **E** lateral **F** dorsal **G** ventral **H** opercular (anterior).

**Figure 12.**
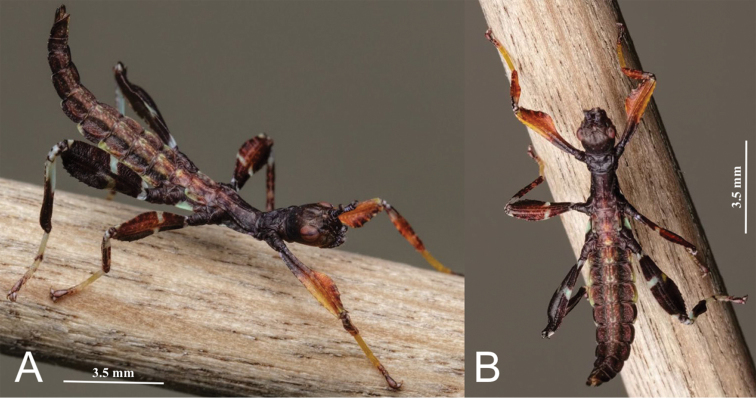
Phyllium (Walaphyllium) monteithi recently emerged first instar nymph showing the unique coloration which may be present in the other members of this new subgenus. Photographs courtesy of Bruno Kneubühler. **A** dorsolateral view **B** dorsal view.

Figs 1 , 3C, D , 8A–F , 9A–D , 10A–C , 11E–H , 12

#### Distribution.

Australia, Queensland: Mt. Lewis, near Julatten (Holotype: QMBA); Garrandunga, Polly Creek (Coll RC); Windsor Tableland, NE Mt. Carbine (Paratype: QMBA); Mt. Windsor, Tableland (Paratype: UQIC); Kuranda (Paratype: ANIC; Coll JHT); Innisfail (Paratype: QMBA); Atherton, Tableland (Paratype: QDPC); Cairns District (Paratype: UQIC); Gadgarra State Forest, nr Lake Tinaroo (Paratype: QDPC).

Records taken from specimens examined and from Brock and Hasenpusch (2003).

#### Discussion.

*Phyllium monteithi* is the most common phylliid species from Australia (the second and only other species being *Nanophyllium australianum* Cumming, Le Tirant, & Teemsma, 2018 which is exceedingly rare). For the Phyllium (Walaphyllium) new subgenus, this is the most commonly encountered species and has been in the phasmid breeding community for numerous years.

Female *Phyllium monteithi* can be differentiated from *Phyllium zomproi* by several morphological features. One is the number of teeth on the stridulatory file of the third antennal segment with 27 to 29 teeth on *P. monteithi* (Fig. 10C) and 48 to 50 teeth noted on *P. zomproi* (Hennemann et al. 2009). There is a notable difference in body size between the two species, with *P. monteithi* a medium sized species ranging from 75.0–76.0 mm and *P. zomproi* a large species ranging from 80.0–86.0 mm in length. Additionally, the tubercles on the thorax of *P. zomproi* are more prominent than those found on *P. monteithi* and the point of the subgenital plate in *P. zomproi* is more pronounced than in *P. monteithi*.

Body size: males: 61.0–64.0 mm, females: 75.0–76.0 mm.

#### Newly hatched nymph coloration.

General color throughout the antennae, head, and thorax is dark brown (Fig. 12). The abdomen is of a similar dark brown color but with pale green and brown muddled in. Margins of abdominal segments II through IV with pale mint green margins versus the other segments which have margins which are of a similar muddled brown color like the base color of the segment. Metanotum lateral margins with a distinct pale mint green patch of color. Base coloration of the legs goes from lightest on the anterior pair to darkest on the posterior pair, with the first pair having a burnt orange color, the middle pair a reddish brown color, and the final pair the darkest with a similar dark brown color found on the rest of the body. Meso- and metafemora with a small transverse white band on the proximal end and a broken white transverse line in the middle of the leg segment. Protibiae are the same burnt orange color as the profemora. Meso- and metatibiae are the same color as their adjoining femora, and both have a white patch of color on the proximal portion. Probasitarsi are a golden yellow and the other basitarsi are cream in color.

### Phyllium (Walaphyllium) lelantos

Taxon classificationAnimaliaPhasmidaPhylliidae

sp. nov.

D5AF963C-D687-5485-A2D3-B1117BFB8654

http://zoobank.org/23FAE495-5290-4671-BE92-9A61439B0BE8

**Figure 13.**
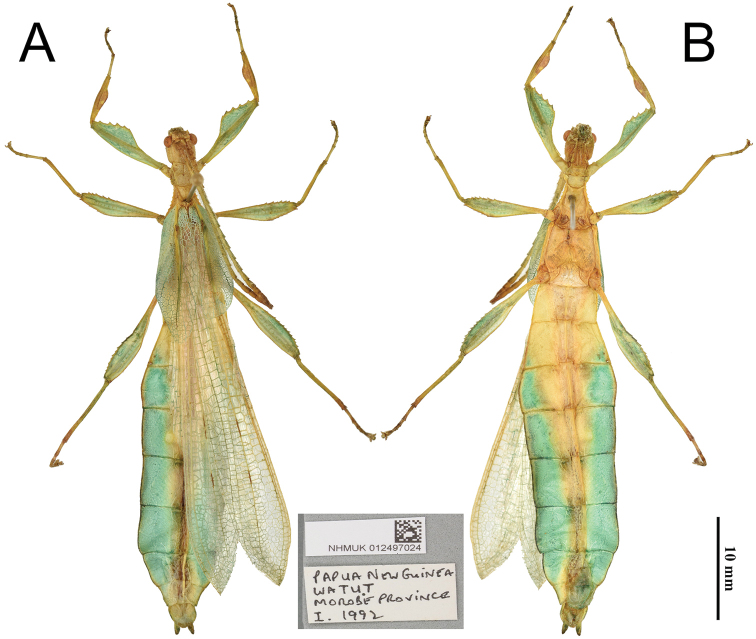
*Phyllium lelantos* sp. nov. holotype male, NHMUK. **A** dorsal **B** ventral. Insert Data labels, “Papua New Guinea, Watut, Morobe Province, I. 1992; NHMUK 012497024”.

**Figure 14.**
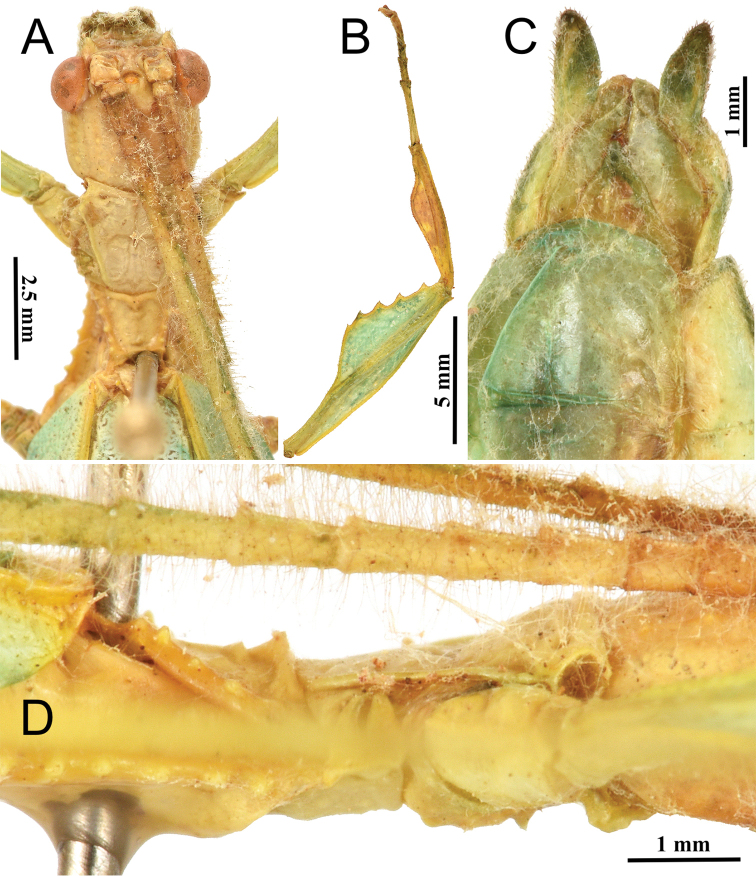
*Phyllium lelantos* sp. nov. holotype male. **A** head and thorax, dorsal **B** front leg, protibiae and profemora, dorsal **C** genitalia, ventral **D** thorax, lateral view.

Figs 3E , 13A, B , 14A–D

#### Type material.

Holotype ♂: Papua New Guinea, Watut, Morobe Province, I.1992. NHMUK 012497024. Deposited in the Natural History Museum, United Kingdom (NHMUK), (Fig. 3E).

#### Discussion and differentiation.

The female and eggs are currently unknown; therefore, differentiation is only given for male morphology. This new species is the smallest within the newly erected subgenus, with the holotype only 53.3 mm long, versus males of *P. monteithi* 61.0–64.0 mm or *P. zomproi* at 79.0 mm (Figs 3B, 3D, versus 3E). An easy morphological feature to differentiate males of the three species is the radial venation of the tegmina. In *P. lelantos* sp. nov. the radial vein is split only once into the first radial and the radial sector; *P. zomproi* has the radial split twice, into the radial sector, the first radial, and the second radial; *P. monteithi* has the radial split more than the others with at least four prominent radials as well as the radial sector (and occasionally a weak but present fifth radial near the wing apex is also present in some specimens). The profemoral exterior lobe is also notably thinner in *P. lelantos* sp. nov. only one or one and a half times wider than the profemoral shaft width (Fig. 14B) versus *P. monteithi* and *P. zomproi* which can have a profemoral exterior lobe width as many as two or two and a half times wider than the profemoral shaft (Figs 6B, 8B).

Morphologically, *P. lelantos* sp. nov. appears to be most similar to *P. monteithi* based on the thorax spination, with the less pronounced mesopleurae tubercles and the anterior prescutum rim with a weakly formed sagittal tubercle (Fig. 14D) versus the prominently raised anterior prescutum rim in *P. zomproi* (Fig. 7E). Another similarity of *P. monteithi* and *P. lelantos* sp. nov. are the terminal abdominal segments with the lateral margins of abdominal segments VIII and IX having straight converging margins, versus *P. zomproi* which has rounded terminal abdominal segments.

#### Description.

**Female and egg**. Unknown.

**Male. *Coloration*.** Coloration description is based on the single preserved holotype specimen. It is expected that live individuals are likely vibrant green in life. Overall coloration green throughout with yellow to tan discoloration in places due to the drying of the specimen. Compound eyes and the four terminal antennae segments are of a rusty brown color darker than the tan on the head or thorax. The rest of the antennae are of a tan to green color. The protibial interior lobe has brown marking throughout most of the surface, this is the only lobe that has brown colorations, the others are a normal green like the rest of the body. The head through to the thorax are more tan than green in the dried specimen but were likely a darker green in life. There are two faint circular eye spots on the fifth abdominal segment with all others lacking markings.

***Morphology.****Head*. Head capsule slightly longer than wide, with a vertex marked by sparse, small evenly sized nodes. Frontal convexity stout with sides that converge to the point which is slightly recurved, not straight; the surface is sparsely covered in thin transparent setae (Fig. 14A). *Antennae*. Antennae consist of 22 segments (including the scapus and pedicellus). The scapus and pedicellus lack setae, the terminal five antennae segments are covered in dense setae that are short, and the remaining antennae segments are covered in long, thin setae which are longer than each segment is wide. Compound eyes are large, notably protruding away from the head capsule and taking up about half of the length of the capsule lateral margins (Fig. 14A). Ocelli are present and well-developed (Fig. 14A). *Thorax*. Pronotum with anterior margin that is slightly concave and lateral margins that are straight and uniformly converging to a straight posterior margin that is about half the width of the anterior rim. Anterior and lateral margins of the pronotum have distinct rims, and the posterior margin lacks a rim (Fig. 14A). Face of the pronotum is marked by a distinct sagittal and transverse furrow meeting at a central pit with the remainder of the surface slightly lumpy in texture. Prosternum is moderately granulose throughout with nodes of even size and slightly uneven spacing as well as a slightly more prominent central node which is more prominent than the others (Fig. 14D). Mesosternum surface wrinkled and marked with nodes throughout. Prescutum anterior margin is wider than the prescutum is long, with lateral margins that converge to the posterior margin that is approximately three-quarters as wide as the anterior rim. Lateral rims with five nodes of slightly varying size but nearly even spacing. Prescutum crest along the sagittal plane nearly bare, with only a single small node present near the anterior rim and one more small one near the posterior, the rest of the sagittal crest lacks features and is just smooth (Fig. 14D). Prescutum anterior margin not particularly prominent, only marked with a single small tubercle (Fig. 14D). Mesopleurae narrow, with margins which are straight, only slightly diverging along their length. Lateral margin marked with six tubercles spread throughout the length almost evenly, with the smallest near the anterior rim and the rest slightly increasing in size as they span towards the posterior (Fig. 14A). Face of the mesopleurae smooth except for two faint divots, one on the anterior third and one on the posterior third. *Wings*. Tegmina short, only extending half way through abdominal segment III. Tegmina venation can only be noted to the best of our ability from the folded wings of the holotype specimen. For the tegmina, the subcosta (Sc) is the first vein and rather long, running smoothly along the wing and terminates just past the midline of the wing length. The radius (R) spans the entire length of the tegmina with the radial sector (Rs) terminating at the wing apex and a single first radius (R1) branching just before the midline and terminating at the margin three quarters of the way through the tegmina length. The media (M) also spans the entire length of the tegmina (as the media anterior MA, which terminates at the wing apex). There are two posterior media veins, the first posterior media (MP1) branches before the midline and meets the cubitus at the wing margin where the first posterior media terminates. The second posterior media (MP2) branches after the first posterior media near the midline and meets with the cubitus near the wing margin. The cubitus (Cu) vein runs along most of the tegmina margin and terminates about three quarters of the way through the tegmina length upon meeting the second media posterior. The first anal (1A) vein terminates upon reaching the cubitus about one-third of the way along the tegmina length. Alae long and well developed in an oval fan configuration, extending to the posterior margin of abdominal segment IX. Alae wing venation cannot be seen fully due to the folded wings, but what can be seen is that there is a fully developed costa (C), subcosta (Sc), and first radius (R1) present along the length of the alae running parallel with each other to the apex of the wing. *Abdomen*. Abdominal segments II through the anterior half of IV gradually and uniformly diverging, posterior half of IV through VII parallel, with each segment only about one and a half times wider than long giving the abdomen a thin boxy appearance. Segments VIII through X converging to the apex but with slightly rounded margins, not perfectly straight. Abdominal segment X has margins which are lined with stout setae, very similar to those which line the cerci margins. *Genitalia*. Poculum starting at the anterior margin of abdominal segment VIII, uniformly broad throughout its length, and ends in a broad slightly rounded apex that slightly passes the anterior margin of segment X (Fig. 14C). Cerci about as long as the vomer, with about half of the length extending from under abdominal segment X. Cerci are slightly cupped and have a surface which is weakly granular. Cerci margins are lined with stout tan setae with those on the exterior margins more prominent than those on the interior margins. Vomer moderately long, extending about three-quarters of the way under segment X with sides that are evenly converging, the single apical point is broad and hooks upwards into the paraproct (Fig. 14C). *Legs*. Profemora exterior lobe slightly wider than half the width of the interior lobe, no small teeth are present but the entire length has short tan setae relatively evenly spaced, and the lobe arcs smoothly along the profemoral shaft without a distinct bend. Profemoral interior lobe roundly triangular without a strong angle and marked with five teeth. The proximal most tooth is smaller than the rest but clearly present, the remaining four teeth are of a similar size and with wide looping spacing except between the distal two which have slightly wider spacing than the others. Exterior mesofemoral lobe arcs end to end with a slight rounded angle in the center, and only on the distal half of that gentle bend are three to five small serrate teeth. The exterior mesofemoral lobe ends in a prominent spur near the mesotibial joint, this spur is larger than any of the preceding small serrate teeth. Interior mesofemoral lobe is slightly narrower than the exterior lobe and lacks a distinct bend, instead hugging the mesotibial shaft. On the distal half only of the interior mesofemoral lobe there are five to six serrate teeth which are larger than the exterior lobes teeth. Metafemoral exterior lobe lacks teeth, is nearly straight, and runs uniformly along the metafemoral shaft. Metafemoral interior lobe slightly wider than the exterior lobe and with nine to ten small serrate teeth throughout slightly more than half of the length. Protibiae lacking an exterior lobe, interior lobe reaching end to end in a smooth scalene triangle, with the broadest portion on the distal end (Fig. 14B). Uniformly throughout the protibial lobe margins are similar stout setae as those found on the profemoral lobes margins. Meso- and metatibiae simple, lacking lobes completely.

#### Etymology.

Noun. Because leaf insects are such cryptic insects so rarely observed, or in this case only known from a single specimen, we felt that the homage of Lelantos the Greek minor Titan of “moving unseen” was fitting. Lelantos’ name is derived from the Greek Ληλαντος (*lêthô*, *lanthanô*, and *lelathon*), meaning “to escape notice”, “move unseen”, or “go unobserved” (Rouse 1942).

#### Distribution.

Only known from the holotype specimen from Papua New Guinea, Morobe Province, Watut (Fig. 4).

#### Measurements of holotype [mm].

Length of body (including cerci and head, excluding antennae) 53.3, length/width of head 3.8/2.8, antennae (but slightly bent so measurement is slightly off) 12.0, pronotum 2.5, mesonotum 1.5, length of tegmina 15.1 (left) and 11.8 (right, which appears to be slightly aberrant and dwarfed), length of alae 38.5, greatest width of abdomen 10.5, profemora 7.4, mesofemora 8.8, metafemora 11.0, protibiae 5.8, mesotibiae 5.7, metatibiae 7.5.

## Biogeography

The Torres Strait is a narrow body of water which separates mainland points of northern Queensland, Australia from southern New Guinea by approximately 130 km (Sands and New 2008). While presently separated, these two regions lay on the Sahul shelf and were connected by a land bridge until roughly 19 to 22 KYA at the end of the last glacial maximum (Clark et al. 2009; Yokoyama et al. 2000). Maps considering potential sea level fluctuations from 17-250 KYA revealed the Torres Strait region remained as a relatively stable land bridge between Australia’s Cape York Peninsula and southern New Guinea (Voris 2000). These past connections across the Torres Strait and the tectonic structure of the region have been used to explain the complex biogeography of modern species in northern Australia and southern New Guinea (Crisp et al. 1995; De Boer and Duffels 1996; Heads 2001; Heads 2014; Grehan and Mielke 2018) and may explain the restricted distribution of Phyllium (Walaphyllium) subgen. nov.

The present status of the Torres Strait as a barrier has been debated for different taxa (Taylor 1972; Sands and New 2008) as it consists of over 150 islands (Green et al. 2010) which could serve as potential habitats. However, these islands may still act as a barrier to the Phylliidae as they consist of dry, bare rock, sandy banks, coral cays, swamps or mangroves, none of which are suitable to the rainforest dwelling Phylliidae. Additionally, recent sea level rise has been accompanied by aridification, with the Cape York Peninsula’s wet forests becoming reduced to mountainous scarps along the eastern coastline since the last glacial maximum (Hope et al. 2004). The most extensive block of present-day rainforests in Cape York are the Kutini-Payamu (Iron Range-McIlwraith Region) which is where the rare *Nanophyllium australianum* Cumming, Le Tirant, & Teemsma, 2018 occurs (Rentz 1988; Adam 1992). This species closely resembles *Nanophyllium pygmaeum* Redtenbacher, 1906, which is known from southern New Guinea near the tip of Cape York, supporting potential descent from New Guinea. Phyllium (Walaphyllium) subgen. nov. has a restricted distribution at each end of the Torres Strait with the northernmost species, *P. lelantos* sp. nov. in the Morobe Province and *P. zomproi* found in the Morobe and Gulf Provinces of Papua New Guinea. The most southern extension is found with *P. monteithi*, which occurs throughout the Wet Tropics of Northeast Queensland. The Wet Tropics consists of the largest continuous area of rainforest in Australia (Adam 1992) and remains as a patch of the rainforests which may have extended across the Sahul shelf, connecting Papua New Guinea and Australia. Future investigation into molecular phylogenies for this clade will help reveal the underlying higher taxonomy within Phylliidae and potential dispersal patterns or evolutionary centers.

## Discussion

While reviewing specimens of this clade we propose herein as *Walaphyllium*, we found that they contained a combination of morphological features which made it unclear to which taxonomic level it should be placed (as a genus or a subgenus). Presently the genus *Phyllium* has several morphologically distinct clades which are treated taxonomically as subgenera (*Phyllium* Illiger, 1798; *Pulchriphyllium* Griffini, 1898; and *Comptaphyllium*Cumming et al. 2019). However, there are other distinct clades within the Phylliidae which are currently classified as genera (*Chitoniscus* Stål, 1875; *Nanophyllium* Redtenbacher, 1906; *Microphyllium* Zompro, 2001; and *Pseudomicrophyllium* Cumming, 2017).

Ultimately, we decided to place this clade as a subgenus of *Phyllium*, but not as a genus of their own due to several morphological factors. First, the two previously described species we here transfer to *Walaphyllium* (*P. monteithi* and *P. zomproi*) have historically been placed within the morphologically diverse *siccifolium* species group of the *Phyllium* subgenus, which concealed their unique combination of features (Hennemann et al. 2009). It wasn’t until we tabulated the morphological diversity of the *Phyllium* subgenera (see Table 1), that we realized that the *Walaphyllium* clade essentially contained a mixture of morphological features which are shared between the *Pulchriphyllium*, *Comptaphyllium*, and/or the *Phyllium* subgenera.

Features which are shared between the *Walaphyllium* and the subgenera *Comptaphyllium* and *Phyllium* are: females with the media and cubitus veins which have spacing between them several times wider than a single vein width (*Pulchriphyllium* has these two veins close together, with a distance less than one vein width apart). On the other hand, a shared feature between the *Pulchriphyllium* and the *Walaphyllium* that separates them from the other subgenera are the eggs, which lack pinnae, have a brittle pitted surface, and have an operculum which is conically raised. No other features were found that were worth noting as unique between clades, either due to variability within subgenera or simply the features were universal among them and not worth noting (see Table 1 for a summary).

The only autapomorphy we were able to identify for the *Walaphyllium* is that the female tegmina have the posterior cubitus branched into the first and second posterior cubitus veins (Fig. 5A), other phylliid females have an unbranched posterior cubitus. We felt that with so few features which are exclusively unique to the *Walaphyllium*, and with a combination of features which link this clade to the other *Phyllium* subgenera, that at this time it is most appropriate to place this clade as a subgenus within *Phyllium*, not as their own genus.

A possible autapomorphy for the *Walaphyllium* is the morphology of the individual teeth on the stridulatory file of the female third antennomere. Unfortunately, there are very few published SEM images of stridulatory files for the different phylliid clades so our observations lack sufficient support to be considered definitive. Friedemann et al. (2012) imaged what they call “*Phyllium siccifolium*” but due to the extreme rarity of that species and incongruous morphological features it is dubious that it is a true *Phyllium siccifolium*. The specimen was only partially imaged and did not note a collection location. Based on the whole antennae image in their figure 4A, which has a ten segmented antennae, the fourth antennal segment which is not short and disk-like, and the number of teeth visible on the stridulatory file, it is possible that the species they actually imaged was *Phyllium philippinicum*Hennemann et al., 2009. Either way, this would represent a member of the *siccifolium* species group of the Phyllium (Phyllium) subgenus and can be used for comparative purposes. These stridulatory file teeth, although not individually imaged, appear in figure 4B of Friedemann et al. (2012) to be raised ovoids with a smooth surface.

Fortunately, members of the other *Phyllium* subgenera have had their stridulatory files imaged with a SEM. The Phyllium (Pulchriphyllium) based on an individual of *Phyllium bioculatum* Gray, 1832 was presented in figure 5 of Bradler (2009) and the Phyllium (Comptaphyllium) clade was imaged on *Phyllium riedeli* Kamp & Hennemann, 2014 in their figure 2E. Both of these subgenera appear to have individual teeth which are raised ovoids with a smooth surface, similar to the Phyllium (Phyllium) subgenus.

In contrast, *P. monteithi*, instead has teeth which are raised ovoids with a distinct central pit at the apex of each individual tooth (Fig. 10B). This feature could possibly be an autapomorphy for the *Walaphyllium* clade but these few examples leave much unknown and no significant conclusions can be drawn from these limited observations.

Our inclusion of SEM images of the *P. monteithi* antennae structure is largely an attempt to illustrate the stridulatory file morphology, to visualize this previously unpublished fine detail, and to demonstrate that there is variable morphology at this level which should be included in future revisionary works. Future SEM visualization projects will image all phylliid clades and several representatives within each clade to allow better morphological comparison and possibly reveal underlying morphological relationships.

Despite the *Walaphyllium* being a small clade of only three species, there are morphological features which suggest the internal relatedness of these species. Based on the spination of the thorax it appears as though *P. monteithi* and *P. lelantos* sp. nov. are likely sister species as evident by their weakly formed prescutum anterior sagittal tubercle (Fig. 14D) and less pronounced mesopleurae tubercles (Fig. 8F), versus *P. zomproi* which has a prominently raised prescutum anterior sagittal tubercle (Fig. 7E) and prominent mesopleurae tubercles (Fig. 7B). Unfortunately the female and egg morphology for *P. lelantos* sp. nov. are unknown at present, and it is possible that these unknowns might hold additional features which illustrate the relationship of species within the *Walaphyllium* clade with more clarity. But with the females and eggs only known for the other two species we cannot at the present draw conclusions as to the internal *Walaphyllium* clade relationships beyond what is suggested by male morphology alone.

These morphologically based observations are currently the compelling evidence to differentiate the *Walaphyllium* from other clades. It is expected that upcoming molecular analyses will help to clarify the higher taxonomy within the Phylliidae, and will reveal if the *Walaphyllium* is a sublineage within the *Phyllium* or if this clade warrants treatment as a separate genus. But, at the present we do not have molecular evidence to suggest the proper placement of this clade, and therefore we place it under the taxonomic umbrella of *Phyllium*.

### Identification key to known males of Phyllium (Walaphyllium) subgen. nov.

Females and eggs are only known for *Phyllium monteithi* and *Phyllium zomproi*, therefore only a key to males is included here. See Brock and Hasenpusch (2003) for a discussion on the morphological differences between *Phyllium monteithi* and *Phyllium zomproi* females and eggs.

**Table:** 

1	Profemoral exterior lobe less than two times the greatest width of the profemoral shaft; tegmina with a radial sector vein (RS) and only one additional radial vein (R1); Papua New Guinea	***P. lelantos* sp. nov.**
–	Profemoral exterior lobe wider than two times the greatest width of the profemoral shaft; tegmina with a radial sector vein (RS) and more than one additional radial vein (two to four additional radials)	**2**
2	Large body size (~79.0 mm); margin of abdominal segments VIII and IX not uniformly converging to the terminal abdominal segment, instead segment VIII is subparallel, and segment IX is strongly rounded, not straight; tegmina with a radial sector vein (RS) and two additional radial veins only (R1 and R2); Papua New Guinea	***P. zomproi***
–	Medium body size (61.0–64.0 mm); margin of abdominal segments VIII and IX with straight margins uniformly converging to the terminal abdominal segment; tegmina with a radial sector vein (RS) and four additional radial veins (R1, R2, R3, and R4); Northeastern Australia	***P. monteithi***


## Supplementary Material

XML Treatment for Phyllium (Walaphyllium)

XML Treatment for Phyllium (Walaphyllium) zomproi

XML Treatment for Phyllium (Walaphyllium) monteithi

XML Treatment for Phyllium (Walaphyllium) lelantos

## References

[B1] Adam P (1992) An Overview of Australian Rainforests. Australian Rainforests. Oxford. Biogeography Series. Oxford University Press, Oxford, 328 pp.

[B2] Bradler S (2009) Die Phylogenie der Stab- und Gespenstschrecken (Insecta: Phasmatodea) “Phylogeny of the stick and leaf insects (Insecta: Phasmatodea)”. Species, Phylogeny and Evolution 2: 3–139. 10.17875/gup2009-710

[B3] Brock PD Büscher TH Baker E (2020) Phasmida SF: Phasmida Species File Version 5.0/5.0. In: Roskov Y., et al. (Eds) Species 2000 & ITIS Catalogue of Life. Species 2000: Naturalis, Leiden. https://www.catalogueoflife.org/col

[B4] Brock PD Hasenpusch J (2003) Studies on the leaf insects (Phasmida: Phylliidae) of Australia. Journal of Orthoptera Research 11: 199–205. 10.1665/1082-6467(2002)011[0199:SOTLIP]2.0.CO;2

[B5] Burt DRR (1932) The venation of the wings of the leaf-insect *Pulchriphyllium crurifolium*. Ceylon Journal of Science, Spolia Zeylanica 17: 29–37.

[B6] Clark PU Dyke AS Shakun JD Carlson AE Clark J Wohlfarth B Mitrovica JX Hostetler SW McCabe AM (2009) The Last Glacial Maximum. Science 325: 710–714. 10.1126/science.1172873 19661421

[B7] Crisp MD Linder HP Weston PH (1995) Cladistic biogeography of plants in Australia and New Guinea: Congruent pattern reveals two endemic tropical tracks. Systematic Biology 44: 457–473. 10.2307/2413654

[B8] Cumming RT Le Tirant S Teemsma S (2018) Northeastern Australia record of *Nanophyllium pygmaeum* Redtenbacher, 1906, now recognized as a new species, *Nanophyllium australianum* n. sp. (Phasmida, Phylliidae). Faunitaxys 6: 1–5.

[B9] Cumming RT Le Tirant S Hennemann FH (2019) A new leaf insect from Obi Island (Wallacea, Indonesia) and description of a new subgenus within *Phyllium* Illiger, 1798 (Phasmatodea: Phylliidae: Phylliinae). Faunitaxys 7: 1–9.

[B10] Cumming RT Bank S Le Tirant S Bradler S (2020) Notes on the leaf insects of the genus *Phyllium* of Sumatra and Java, Indonesia, including the description of two new species with purple coxae (Phasmatodea, Phylliidae). ZooKeys 913: 89–126. 10.3897/zookeys.913.49044 32132850 PMC7044250

[B11] De Boer AJ Duffels JP (1996) Historical biogeography of the cicadas of Wallacea, New Guinea and the West Pacific: A geotectonic explanation. Palaeogeography, Palaeoclimatology, Palaeoecology 124: 153–177. 10.1016/0031-0182(96)00007-7

[B12] Dixon RM (1972) The Dyirbal Language of North Queensland. Cambridge University Press, London, 420 pp 10.1017/CBO9781139084987

[B13] Friedemann K Wipfler B Bradler S Beutel RG (2012) On the head morphology of *Phyllium* and the phylogenetic relationships of Phasmatodea (Insecta). Acta Zoologica 93: 184–199. 10.1111/j.1463-6395.2010.00497.x

[B14] Green D Alexander L McInnes K Church J Nicholls N White N (2010) An assessment of climate change impacts and adaptation for the Torres Strait Islands, Australia. Climatic Change 102: 405–433. 10.1007/s10584-009-9756-2

[B15] Grehan JR Mielke CGC (2018) Evolutionary biogeography and tectonic history of the ghost moth families Hepialidae, Mnesarchaeidae, and Palaeosetidae in the Southwest Pacific (Lepidoptera: Exoporia). Zootaxa 4415: 243–275. 10.1007/s10584-009-9756-2 30313621

[B16] Griffini A (1898) Intorno al *Phyllium geryon* Gray. Bollettino dei Musei di Zoologia ed Anatomia comparata della Royal Università di Torino 8: 1–4. https://biodiversitylibrary.org/page/11257682 10.5962/bhl.part.27225

[B17] Größer D (2001) Wandelnde Blätter. Ein Katalog aller bisher beschriebenen Phylliinae-Arten und deren Eier mit drei Neubeschreibungen. Frankfurt am Main, Germany (Edition Chimaira), 119 pp.

[B18] Heads M (2001) Birds of paradise, biogeography and ecology in New Guinea: A review. Journal of Biogeography 28: 893–925. 10.1046/j.1365-2699.2001.00600.x

[B19] Heads M (2014) Biogeography of Australasia: A Molecular Analysis. Systematic Biology 64: 163–166. 10.1093/sysbio/syu074

[B20] Hennemann FH Conle OV Gottardo M Bresseel J (2009) On certain species of the genus *Phyllium* Illiger, 1798, with proposals for an intra-generic systematization and the descriptions of five new species from the Philippines and Palawan (Phasmatodea: Phylliidae: Phylliinae: Phylliini). Zootaxa 2322: 1–83. 10.11646/zootaxa.2322.1.1

[B21] Illiger JKW (1798) Verzeichnis der Käfer Preussens. Johann Jacob Gebauer, Halle, 510 pp https://biodiversitylibrary.org/page/52579286

[B22] Kamp TVD Hennemann FH (2014) A tiny new species of leaf insect (Phasmatodea, Phylliidae) from New Guinea. Zootaxa 3869: 397–408. 10.11646/zootaxa.3869.4.4 25283926

[B23] Key KHL (1970) Ch. 22, Phasmatodea (Stick-insects). In: CSIRO (Ed.) The Insects of Australia, A textbook for students and research workers, 348–359. Melbourne University Press, Melbourne.

[B24] Key KHL (1974) Phasmatodea (Stick-insects). In: CSIRO (Ed.) The Insects of Australia. Supplement 1974, 48–49. Melbourne University Press, Melbourne.

[B25] McKeown KC (1940) Australian Insects. VIII, Orthoptera: 3. Stick and leaf insects. The Australian Museum Magazine 7: 125–130.

[B26] Monteith GB (1978) The First Male Leaf Insect from Australia. The Entomological Society of Queensland 5: 138–139.

[B27] Monteith GB (1971) Leaf Insects from Australia. The Entomological Society of Queensland 78: 14–15.

[B28] Musgrave A (1942) An Interesting New Guinea Phasmid. The Australian Museum Magazine 7: 414.

[B29] Patz E (1991) ‘Djabugay.’ The Handbook of Australian languages, Vol. 4. Oxford University Press, Oxford, 314–331.

[B30] Poitout F (2007) Dictionnaire étymologique des noms scientifiques des Phasmes (Phasmatodea). L’Association PHYLLIE, Paris, France, 1–702.

[B31] Ragge DR (1955) The wing-venation of the Order Phasmida. The Transactions of the Royal Entomological Society of London 106: 375–392. 10.1111/j.1365-2311.1955.tb01272.x

[B32] Rentz DCF (1988) *Nanophyllium pygmaeum* Redtenbacher (Phasmatodea: Phylliidae: Phylliinae), A Leaf Insect Recently Recognized in Australia. Australian Entomological Magazine 15: 3–5.

[B33] Rouse WHD (1942) Nonnos Dionysiaca, with an English translation by W. H. D. Rouse. Harvard University Press, Cambridge, Massachusetts, United States, 517 pp.

[B34] Sands DPA New TR (2008) Conservation status and needs of butterflies (Lepidoptera) on the Torres Strait Islands. Insect Conservation and Islands 12: 131–138. 10.1007/978-1-4020-8782-0_11

[B35] Schneeberg K Bauernfeind R Pohl H (2017) Comparison of cleaning methods for delicate insect specimens for scanning electron microscopy. Microscopy Research and Technique 80: 1199–1204. 10.1002/jemt.22917 28802096

[B36] Simon S Letsch H Bank S Buckley TR Donath A Liu S Machida R Meusemann K Misof B Podsiadlowski L Zhuo X Wipfler B Bradler S (2019) Old World and New World Phasmatodea: Phylogenomics Resolve the Evolutionary History of Stick and Leaf Insects. Frontiers in Ecology and Evolution 7: 1–14. 10.3389/fevo.2019.00345

[B37] Smith WA (1966) A record of *Phyllium* (Phasmida: Phyllidae) from Queensland. Journal of the Entomological Society of Queensland 5: 45.

[B38] Taylor RW (1972) Biogeography of insects of New Guinea and Cape York Peninsula. In: Walker D (Ed.) Bridge and barrier: the natural and cultural history of Torres Strait.Research School of Pacific Studies, Australian National University, Canberra, 213–230.

[B39] Yokoyama Y Lambeck K Deckker PDe (2000) Timing of the Last Glacial Maximum from observed sea-level minima. Nature 406: 1998–2001. 10.1038/35021035 10963593

